# Cognitive behavioral intervention via a smartphone app for non-professional caregivers with depressive symptoms: study protocol for a randomized controlled trial

**DOI:** 10.1186/s13063-018-2793-2

**Published:** 2018-07-31

**Authors:** Fernando L. Vázquez, Ángela Torres, Olga Díaz, Mario Páramo, Patricia Otero, Vanessa Blanco, Lara López

**Affiliations:** 1 Department of Clinical Psychology and Psychobiology, University of Santiago de Compostela, Santiago de Compostela, Spain; 2 Department of Psychiatry, Radiology, Public Health, Nursing and Medicine, University of Santiago de Compostela, Santiago de Compostela, Spain; 3 Department of Psychology, University of A Coruña, A Coruña, Spain; 4 Department of Evolutive and Educational Psychology, University of Santiago de Compostela, Santiago de Compostela, Spain

**Keywords:** Non-professional caregivers, App, Smartphone, Depression, Prevention, Adherence to the intervention, Study protocol

## Abstract

**Background:**

Although major depression is a frequent disorder in non-professional caregivers and there are effective psychological interventions to prevent it, caregivers have difficulty accessing them. Interventions for depression applied through an app could improve accessibility; yet, to date, adherence to such interventions has been low. The objectives of this study are to (1) evaluate the efficacy of a cognitive behavioral depression prevention intervention administered through a smartphone app with and without telephone conference calls, (2) analyze the mediators of the change in the incidence of depression and depressive symptoms, and (3) assess adherence and satisfaction with the interventions.

**Methods:**

A randomized controlled clinical trial will be conducted. Caregivers with elevated symptoms will be randomly assigned to a cognitive behavioral intervention administered by a smartphone app (CBIA) group, a CBIA plus telephone conference calls (TCCs) group (CBIA + TCC), or an attention control group. Each condition will consist of approximately 58 participants. Both interventions will be administered in five modules through a smartphone app and the CBIA + TCC group will receive additional TCCs in group format (four sessions of 30 min each). Trained blind assessors will conduct pre-treatment, post-treatment and follow-up assessments at 1, 3, 6, and 12 months.

**Discussion:**

This study will provide evidence of the efficacy of a cognitive behavioral intervention to prevent depression in caregivers with elevated depressive symptoms administered through a smartphone app and the impact of feedback applied through conference calls to increase program adherence and efficacy. If the results were favorable, it would mean that we have developed a more effective, accessible, and clinically useful preventive depression intervention than the currently available ones for many present and future caregivers.

**Trial registration:**

ClinicalTrials.gov: NCT03110991. Registered 5 April 2017.

**Electronic supplementary material:**

The online version of this article (10.1186/s13063-018-2793-2) contains supplementary material, which is available to authorized users.

## Background

In European countries, apprximately 54.5 million people are caregivers of a loved one in a situation of dependency [1]. However, staying in this situation for a prolongued period can affect their physical and mental health [2]. In the context of mental health, major depression is the most frequent mental disorder in this population, with a prevalence of 8.9% [3]. These figures are highly relevant because depression is associated with impaired function [4] and may interfere with the quality of care of the dependent [5].

Interventions to prevent depression may prevent or reduce the occurrence of these cases of depression. Specifically, prevention interventions aimed at caregivers with high depressive symptoms but who have not yet developed clinical depression (i.e., indicated prevention) have the greatest empirical evidence to date [6–9]. One of these interventions, evaluated by Vázquez et al. [8, 9], was developed based on Lewinsohn et al.’s [10] multifactorial integrator model of depression. This intervention found a significant reduction in the incidence of depression and depressive symptoms in the cognitive behavioral intervention group compared to the usual care control group at post-treatment [8] and after 12 months of follow-up [9]. Moreover, the changes found in relation to depressive symptoms were clinically significant [11]. Although these results are promising, the intervention was applied face-to-face, which may limit its impact on public health since this format presents a series of barriers that make it less accessible to caregivers. Many caregivers find it difficult to attend interventions due to lack of time, displacement problems, not having a caregiver substitute for their dependent during their absence, lack of mental health services, or stigmatization.

These accessibility issues could be easily mitigated through information and communication technologies, among which that with the greatest potential is the mobile phone. Mobile phones are owned by more than 80% of the world’s population [12], they are portable and can serve as a platform for a variety of apps thanks to its accessibility to programming and internet access (smartphones). However, there are currently only two randomized controlled trials that have evaluated interventions for the treatment of depression through an app, neither of which was aimed at preventing depression in caregivers and only showing partial improvements in the reduction of depressive symptoms. More specifically, Arean et al. [13] found no significant difference at the post-treatment and 1-month follow-up between a cognitive training intervention, another intervention based on problem-solving therapy, and an attention control group where participants received therapeutically inactive information on health (Health Tips). At the 3-month follow-up, participants in both interventions showed higher remission rates compared to controls (50% and 49% vs. 32%), but only those with moderate depressive symptomatology in the problem-solving therapy group showed less depressive symptoms than the control group (*d* = 0.76). In addition, Ly et al. [14] did not find any post-treatment differences between a program based on behavioral activation and another based on mindfulness. At the 6-month follow-up, the behavioral activation intervention was more effective for patients with greater initial severity of depression (*d* = 0.47), whereas the mindfulness intervention was more effective for patients with less severity (*d* = 0.98).

The limitations in both studies included problems of adherence to the interventions. Dropout rates were high, ranging from 14.8% [14] to 31.6% [13]; in the study by Arean et al. [13], 57.9% did not even download the assigned intervention app and the level of compliance with homework assignments was not reported. These issues are fundamental because individuals who leave the intervention show worse results and less satisfaction with the therapy, and when evaluating the efficacy of interventions, high dropout rates can lead to bias due to attrition, limiting the generalizability of the results [15]. In addition, homework completion is a significant predictor of therapy outcomes in various populations and interventions [16], including depression prevention interventions in the caregiver population [17]. This shows that when more homework is completed, the therapy results are better, leading to a greater reduction of depressive symptoms [17]. Complementing the interventions implemented through smartphones with a telephone conference call (TCC) to give feedback could help solve these problems. In behavioral skills training procedures, feedback is specifically defined as delivery of praise for correct performance of a target behavior and further instruction following incorrect performance [18].

The objectives of the present study are to (1) assess the efficacy of a cognitive behavioral intervention for indicated prevention of depression in informal caregivers, administered through a smartphone app (CBIA) with and without TCCs with respect to a care control group, (2) analyze the mediators of change in depressive symptoms, and (3) assess adherence and satisfaction with the interventions. As a central hypothesis, both interventions are expected to significantly reduce the incidence of depressive episodes and depressive symptoms compared to the control group at post-treatment and at 1-, 3-, 6-, and 12-month follow-up. Secondary hypotheses are (1) that the change in reinforcement and in negative automatic thoughts will mediate the effects of both interventions and (2) that the participants receiving CBIA + TCC will show greater adherence and satisfaction with the intervention than those who receive CBIA alone.

## Methods

### Design

A randomized controlled clinical trial will be conducted to study the efficacy of a cognitive behavioral intervention for non-professional caregivers with elevated depressive symptoms administered through a smartphone app with and without TCCs. Specifically, eligible participants will be randomly assigned to one of three conditions, as follows: (1) CBIA; (2) CBIA + TCC; and (3) an attention control group (ACG).

**Fig. 1:**
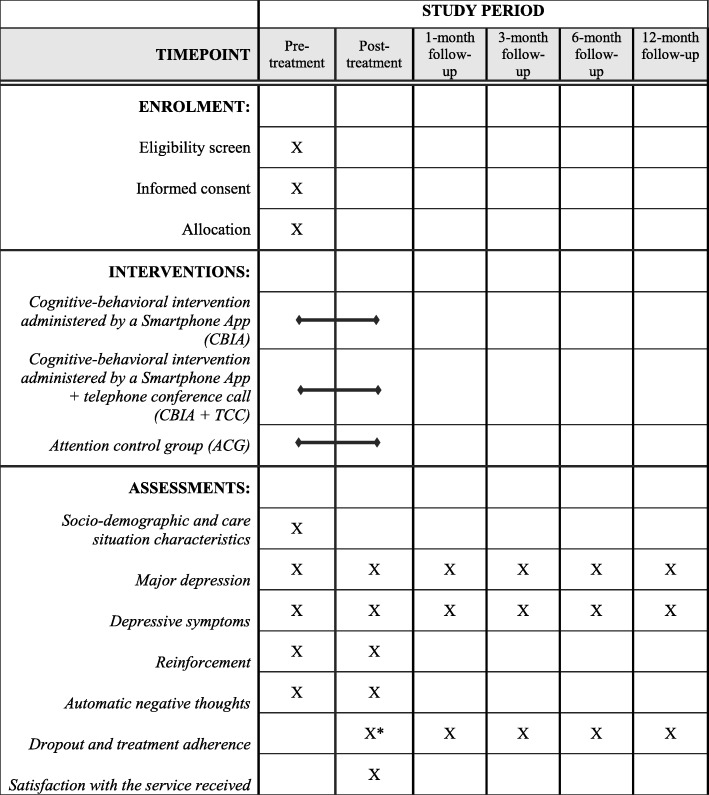
SPIRIT Figure. Phases of the randomized controlled trial. Note: * During the intervention and at post-treatment

The study phases are shown in Fig. 1. There will be six measurement points in the three groups (i.e., pre-treatment, post-treatment, and follow-up at 1, 3, 6, and 12 months). After the baseline assessment (pre-treatment), and once caregivers who meet the eligibility criteria are selected and interventions are administered, a post-treatment evaluation and four follow-ups will be performed (at 1, 3, 6, and 12 months). To minimize the loss of participants, we will follow the strategies recommended by Grady et al. [19], such as selecting participants who are likely to adhere to the intervention, obtaining various means to contact the participants (address, telephone, email), and making the intervention easy.

### Sample size

Based on a previous study of indicated prevention of depression in caregivers [9], which involved the same program that will be evaluated in the present study (albeit in person), we estimate that a sample size of 49 participants per group (98 in total) would be sufficient to detect an 18.6% difference in the incidence rates of major depression episodes between the experimental group and the control, assuming a two-tailed test α of 0.05 and a power (1 – β) of 0.80. However, considering an attrition rate of approximately 15% based on studies that have evaluated the use of interventions for the treatment of depression applied through an app [14], we estimate that the number of participants per group should be 58, resulting in a final sample size of 174.

### Participants, recruitment, and eligibility criteria

Participants will be recruited from the population of non-professional caregivers of persons in a situation of dependency officially recognized by the Regional Government of Galicia. Galicia is a region in the northwest of Spain with an area of 29,434 km^2^ and a population of 2,730,337.

It has been previously determined that between 39% and 44% of caregivers present with elevated depressive symptoms [7, 8] and 8.9% have had a major depressive episode [3]. As a result, to achieve the estimated sample of about 174 participants, we would need to select approximately 532 caregivers for screening prior to verification of eligibility criteria.

Caregivers will be contacted through a letter inviting them to participate in the study and asking them to return a sealed postcard if they do not wish to be contacted again. Caregivers who do not send the card will be contacted by phone. At that time, a brief description of the study will be given, and those who are interested will receive a brief screening call to evaluate their depressive symptoms, the diagnostic criteria for major depressive episodes, and the eligibility criteria. Those who meet the initial selection criteria will be invited to participate in a full evaluation through the app.

To be included in the study, participants must meet the following inclusion criteria: (1) be a non-professional caregiver of a dependent family member whose dependency is officially recognized by the Regional Government of Galicia; (2) have a smartphone; (3) present a clinically significant symptomatology defined as a score equal to or greater than 16 in the Spanish version of the Center for Epidemiologic Studies Depression Scale (CES-D [20]); (4) not meet the diagnostic criteria for a major depressive episode according to the Diagnostic and Statistical Manual of Mental Disorders (DSM-5) [21]; (5) not have a history of major depression; (6) commit to participating in all evaluations; and (7) provide written informed consent. Participants who meet the following criteria will be excluded: (1) receiving psychological or psychopharmacological treatment in the last 2 months; (2) have other disorders that may act as confounding variables (e.g., symptoms due to substance use); (3) have severe psychological or medical disorders requiring immediate intervention (e.g., suicidal ideation) or that make the study impossible (e.g., significant cognitive impairment); (4) that the person in a situation of dependence has a severe or terminal prognosis within the next 14 months; or (5) that in the next 14 months they plan to move or to institutionalize the dependent they were caring for.

### Randomization

Those participants who meet the eligibility criteria will be randomly assigned to one of the three conditions, CBIA, CBIA + TCC, or ACG, by a statistics expert not related to the study using a table of random numbers generated by a computer. The sequence of randomization will be communicated to the researchers by means of sealed numbered envelopes, one for each participant, with instructions to use them in numerical order. Due to the nature of the intervention, it is not possible to blind the participants for allocation status.

### Interventions

We have developed a protocol intervention and manualized each intervention to increase internal validity. In the CBIA group, all participants will receive the same intervention content through a smartphone app. In the CBIA + TCC group, in addition to the aforementioned intervention, the intervention will be applied by psychologists (masters or doctoral-level degree) who will be trained for the administration of the intervention by two professionals with more than 20 years of experience in cognitive behavioral therapy. They will receive approximately 35 h of training consisting of theoretical and practical seminars and role-playing exercises. In this group, TCC sessions will be recorded. The professionals who will participate in therapists’ training will assess the degree of adherence to the manuals and the ability to apply the interventions, and will monitor the therapists weekly.

#### Cognitive-behavioral intervention administered through a smartphone app (CBIA)

We have adapted an indicated prevention intervention of depression based on the multifactorial model of Lewinsohn et al. [10] that has proved its efficacy in previous face-to-face studies [8, 9] and in conference telephone format [22] for application through a smartphone app. This cognitive behavioral intervention consists of five modules to be performed in approximately 5 weeks. In module 1, we will explain the concept of depression, the need for active coping with depressive symptoms, and we will train participants in diaphragmatic breathing, monitoring mood and self-reinforcing techniques. Module 2 will be focused on how pleasant activities affect mood and developing a plan for introducing such activities in the participants’ daily lives with the help of behavioral contracts. In module 3, we will address how thoughts affect mood and participants will be trained on techniques to manage thoughts. In module 4, we will explain how social contacts affect mood, give strategies for assertive communication, and encourage participants to increase their social contacts. In module 5, participants will review everything they have learned throughout the intervention and focus on relapse prevention.

#### Cognitive behavioral intervention administered through a smartphone app + telephone conference call (CBIA + TCC)

This group will receive, in addition to CBIA as described above, a telephone contact in group format (in groups of five caregivers approximately), using a conference system, for five weekly 30-min sessions. Group rules will be explained in the first session and positive or corrective feedback will be administered through the five sessions after revising the performed homework [18]. Positive feedback consists of providing information on the correct implementation of the intersessional tasks and reinforcement, and corrective feedback involves identifying those tasks that have not been adequately carried out and suggesting relevant changes to improve performance. We follow Miltenberg’s guidelines [18] to improve the effectiveness of feedback, namely that (1) it should be given immediately after the target behavior is performed; (2) it should contain praise or other reinforcement for doing the behavior correctly and, if the behavior is incorrect, some type of praise should be given for trying; (3) praise must be descriptive, focusing on how the behavior has been performed; (4) corrective feedback should not be negative and the performance of the participant should not be described as wrong or mistaken (it is better to focus on how to improve performance); (5) always praise some aspect of the behavior before providing corrective feedback; and (6) provide corrective feedback on one aspect of behavior at a time. Participants will also be encouraged to support each other in the process of change.

#### Attention control group (ACG)

Participants in the control group will receive information on depression equal to the CBIA group in duration (five modules), extension, and mode of administration (via a smartphone app). Participants in this group will receive therapeutically inactive information about depression (e.g., what is depression, prevalence, causes, symptoms) and healthy habits.

### Outcomes

As shown in Table 1, we will collect information on the sociodemographic characteristics of caregivers and the care situation, major depressive episodes and other mental disorders, depressive symptoms, mediating variables (environmental reinforcement and negative thoughts), drop out, adherence, and satisfaction with the intervention. The self-administered instruments will be completed by the participants through the app. Hetero-administered instruments will be administered through the telephone by trained interviewers who will be blind to the objectives of the study, the interventions that will be administered, and the randomization to the different groups. The training of the evaluators will be carried out by two study researchers with more than 20 years of experience in evaluation, and will consist of 15 h of theoretical and practical seminars and role-playing about the measurement instruments and the evaluation strategies.

**Table 1:** Overview of measures

Instrument	Format
Participant characteristics	
Sociodemographic and care situation characteristics	Self-administered
Primary outcome	
Major depression: SCID-5-CV	Hetero-administered
Secondary outcomes	
Depressive symptoms: CES-D	Self-administered
Reinforcement: EROS	Self-administered
Automatic negative thoughts: ATQ	Self-administered
Dropout and treatment adherence	Hetero-administered
Satisfaction with the service received: CSQ-8	Self-administered

*SCID-5-CV* Structured Clinical Interview for DSM-5 Disorders, Clinician Version, *CES-D* Center for Epidemiologic Studies Depression Scale, *EROS* Environmental Reward Observation Scale, *ATQ* Automatic Thoughts Questionnaire, *CSQ-8* Client Satisfaction Questionnaire

#### Sociodemographic characteristics and care situation

These will be evaluated through the Characteristics and Status of Caregiver Questionnaire used in previous studies [8, 9]. It records the sociodemographic characteristics of the non-professional caregivers (sex, age, marital status, social class, educational level, main activity) and the care situation (relationship with the person cared for, age and sex of the dependent, disease of the dependent, time caring for their relative and daily hours dedicated to the care).

#### Primary outcome measure: major depression

The presence of a major depressive episode will be evaluated with the Structured Clinical Interview for DSM-5^®^ – Clinician Version (SCID-5-CV; [23]). This provides diagnoses of DSM-5 and must be administered by a clinician. It includes the most common disorders in clinical practice, namely depressive disorder, bipolar disorder, schizophrenia and other psychotic disorders, substance use disorders, anxiety disorders, obsessive-compulsive disorder, post-traumatic stress disorder, attention deficit hyperactivity disorder, and adaptive disorders, and it allows the screening of 17 additional disorders. For the pre-treatment evaluation, the entire interview will be used, while in the following evaluations only the module corresponding to the major depressive episode will be used. This interview has been applied face-to-face and telephonically [24]. Interobserver reliability (Kappa) ranges from 0.70 to 1.00.

#### Secondary outcome measures

##### Depressive symptoms

Depressive symptoms will be evaluated through the CES-D self-report scale [25] (Spanish version of Vázquez et al. [20]). It is a 20-item scale in which individuals report the frequency with which they have experienced each symptom during the previous week. Each of the items is evaluated on a Likert scale of four response options, with a range from 0 (rarely or none of the time) to 3 (most of the time). The total score range expands from 0 to 60, where a higher score corresponds to a greater depressive symptomatology. The internal consistency ranges from 0.85 to 0.90, with the Spanish version having a consistency of 0.89.

##### Reinforcement

In order to evaluate the reinforcement of the environment we will use the self-reported Environmental Reward Observation Scale (EROS) [26] (Spanish version of Barraca and Pérez-Álvarez [27]). It consists of 10 items in which the participant evaluates the degree of positive reinforcement received contingently from their environment on a Likert scale ranging from 1 (strongly disagree) to 4 (strongly agree). The total score ranges from 10 to 40, with a higher score indicating greater positive reinforcement. The internal consistency of the Spanish version is 0.86.

##### Automatic negative thoughts

Negative automatic thoughts will be evaluated through the Automatic Thoughts Questionnaire (ATQ) [28] (Spanish version of Otero et al. [29]). This is a 30-item self-report questionnaire in which participants must indicate, for each item, the frequency with which they experienced a series of thoughts during the prior week, in a Likert scale ranging from 1 (never) to 5 (always). The total score range extends from 30 to 150, where a higher score indicates more negative thoughts. Its internal consistency is 0.96.

##### Dropout and treatment adherence

A record will be kept of the dropouts produced in each group throughout the study. In addition, in the interventions, a record of the number of modules completed and the fulfillment of the intersessional tasks by each caregiver will be made.

##### Satisfaction with the service received

The satisfaction of the participants with the service received once the interventions are completed will be evaluated with the Client Satisfaction Questionnaire (CSQ-8) [30] (Castilian Spanish version of Vázquez et al. [31]). It is a self-reported scale of eight items and four possible answers, with a score ranging from 8 to 32, where a higher score implies a greater satisfaction with the service received. Its internal consistency ranges from 0.80 to 0.93 [32], with the Spanish version having a consistency of 0.80.

### Data management

Personal data (identifying information) and clinical data will be stored separately. The participant files will be stored in numerical order in a safe place. They will be kept for 5 years after completion of the study. All data will be entered into a database, in which individuals cannot be identified. Range checks and consistency checks against data already stored in the database will be made. All evaluation instruments, audio recordings, and hardware related to the study data will be kept in locked cabinets. Access to study data will be restricted, with a password system that only researchers will know, used to control access. A backup of the original database (primary database) will be performed twice a month. All reports and publications derived from the study will be prepared in such a way that no individual can be identified.

### Statistical analyses

We will use the statistical package SPSS for Windows (version 21.0) for the analysis of the data. All analyses will be performed according to the intent-to-treat principle. If participants drop out from the study, the lost values ​​will be imputed using multiple imputation [33].

To analyze the incidence of depressive episodes in each of the measurement times (post-treatment, 1, 3, 6, and 12 months of follow-up), a global χ^2^ test will be performed to compare the three groups. If the differences are statistically significant (*p* < 0.05), pairwise comparisons will be performed using logistic regression. The relative risk and the required number of patients to be treated will be calculated following the formulas proposed by Guyatt et al. [34]. In addition, the time it will take participants to undergo a major depressive episode will be analyzed using a survival analysis. The analysis of the effect of the interventions on the outcome variable ‘depressive symptoms’ will be performed using two-way ANOVA with repeated measures. The analyses related to the effects of moderation or mediation will be carried out as per the recommendations of Baron and Kenny [35]. The model to evaluate moderation will include, as an independent variable, the type of treatment, the moderating potential, and the interaction between the two. An optimal combined moderator will also be sought according to Kraemer’s recommendations [36]. For the analysis of the mediation, we will perform adjustment of three regression equations to assess the effect (1) of the predictor variable (amount of treatment) on the dependent variable (change in depressive symptomatology after the intervention), (2) of the predictor variable in the mediator potential of the predictor variable (change in reinforcement and negative thoughts), and (3) of the mediator potential of the dependent variable, controlled by the predictor variable. Dropouts and adherence to interventions will be analyzed using a χ^2^ test to compare the percentage of dropouts and using an independent *t* test to compare the number of modules completed and the register of the intersessional tasks. Likewise, the level of satisfaction with the interventions will be assessed using the CSQ-8 through a frequency analysis and descriptive statistics, and both active conditions will be compared at post-treatment using an independent *t* test.

### Monitoring

A Data Monitoring Committee (DMC) will be established to monitor and guarantee the correct execution of the study, which will be independent of the organizers of the study and can order an independent audition once a year. The steering committee, led by the principal investigator, will follow the principles of good clinical practice, including quality control of the clinical protocol, data management, and team meeting organization. A confidential annual report on the development of the trial will be sent to the DMC.

A pilot study will be conducted to assess the feasibility of the study. Any significant protocol modification that may affect the performance of the study, the potential benefit to or safety of the patient, including significant changes in study design, population, sample size, or study procedures, will require a formal amendment to the protocol, which will have to be approved by the Bioethics Committee prior to its implementation.

In addition, an independent statistician will conduct a preliminary analysis after the pilot study and when 50% of patients have been randomized and have completed follow-up. The statistician will inform the independent DMC, who will have access to all data and will discuss the results of the analysis with the steering committee at a joint meeting. The steering committee will then decide on the continuation of the trial and report to the Bioethics Committee. Any solicited or spontaneous reported adverse event through the study will be notified to the steering committee, who will take the necessary actions.

### Ethics, consent, and permissions

The human rights and the dignity of the study participants will be protected in accordance with the Declaration of Helsinki. The study procedures have been approved by the Bioethics Committee of the University of Santiago de Compostela (Spain). The confidentiality of all participants will be guaranteed. Participants will have to give their informed consent (first verbally by telephone followed by written consent by mail). Participation will be completely voluntary, without any kind of incentive (economic or other).

During the study, if a caregiver meets the criteria for the diagnosis of a major depressive episode, the individual will be contacted by phone to explain what is happening to him or her and referred to a center where he or she can receive personal treatment (e.g., a primary care center, a mental health service), discontinuing the study. After completing the study, any requested ancillary post-trial care will be managed by the steering committee, who will make referrals to mental health services if necessary.

## Discussion

This study will evaluate the efficacy of a brief cognitive behavioral intervention of indicated prevention of depression administered through a smartphone app with and without TCCs. The intervention will be adapted from a previous study by Vázquez et al. [8, 9]. Based on the results of this previous study, we expect to find a significant reduction in the incidence of depression and depressive symptoms in both intervention groups compared to the control group.

The development of this intervention follows the NICE clinical practice guidelines, which recommend computerized cognitive behavioral therapy and self-help resources (such as the app) for the treatment of people with mild to moderate depression [37]. In addition, using the app to manage the psychological intervention as an alternative to traditional face-to-face programs will increase accessibility to mental health services and increase the tools that professionals have to reach a larger number of people. This is in accordance with the recommendations of the National Institute of Mental Health Psychosocial Intervention Development Workgroup [38] and the New Freedom Commission on Mental Health [39]. The advantages of the intervention being administered through an app, and which increase its accessibility, include anonymity, savings in costs and travel times, the possibility of receiving the intervention anywhere and at any time (being able to receive it at home), without waiting nor the need to make appointments, and being able to review materials as often as necessary, at one’s own pace and with real-time tracking [40].

On the other hand, this study proposes a possible solution to the problem of lack of adherence found in the existing tests of psychological interventions for the treatment of depression administered through an app [13, 14]. Specifically, we propose to complement the intervention program with group conference calls led by a therapist. In Internet-administered interventions, it has been found that adding regular telephone contact increases adherence substantially [41]. In addition, high levels of adherence to the intervention were found in a study [22] in which a cognitive behavioral intervention and a behavioral activation intervention applied by TCC, such as that proposed in this study, were conducted. In that study, average attendances were 4.6 and 4.2 of 5 sessions, respectively, and average completed homework assignments were 14.7 of 18 and 9.5 of 12. This study also showed a low percentage of dropouts (6.6%) at post-treatment, similar to that found at the same time point in a face-to-face intervention [8]. All this suggests that human contact is vital to achieve high levels of commitment to a non-face-to-face psychological intervention.

In addition, the fact that telephone contacts are grouped can save costs and introduce social support, the latter of which is an important strategy in behavior change [42]. Finally, the use of feedback as a psychological technique administered in these telephone contacts, following the guidelines recommended by Miltenberg [18], brings rigor and solidity to the intervention protocol, facilitates its replication, and reinforces the learning of the cognitive behavioral skills trained during the intervention, favoring the expected results.

Among the strengths of this clinical trial are the specification of the level of prevention and the systematic selection of the participants according to this, the prior estimation of the sample size, the randomizing by means of an accepted method, the concealment of the random assignment, the application of an intervention based on a theoretical model of depression with proven efficacy in previous clinical research, the evaluation of adherence to the protocol, and the blind evaluation of the results by trained professionals, as well as follow-ups of up to 12 months. For the evaluation of the results, we will use validated instruments with recognized psychometric properties (see [43]). The incidence of depression as a primary outcome will be assessed with the SCID-5-CV, which has been used as the gold standard for DSM-5 diagnoses [44]. Depressive symptoms as a secondary outcome will be evaluated with CES-D, which is the most widely used instrument for assessing depressive symptoms in the caregiver population [45]. In addition, the study will be performed in the community of caregivers and thus its results have a high level of generalizability.

Although there is currently a rapid proliferation of mental health apps in the commercial field, there are very few that have been shown to be effective [46]. This study can reduce the dangers associated with the lack of quality control of currently available apps, providing an evidence-based intervention to prevent depression.

In conclusion, this study will provide information on the efficacy of the intervention to prevent depression in non-professional caregivers, exploring the use of alternative formats to increase the accessibility of therapies and assessing a technique to improve the adherence and efficacy of the intervention. The results of this study will benefit a large number of present and future caregivers. In addition, it has significant implications for public health in the context of the high social and economic costs of depression [47].

### Trial status

Recruitment start: September 28, 2018.

Study completion: December 29, 2019.

## Additional file

Additional file 1: SPIRIT 2013 Checklist: Recommended items to address in a clinical trial protocol and related documents*. (DOC 121 kb)

## Abbreviations

ACG: Attention control group
ATQ: Automatic Thoughts Questionnaire
CBIA: Cognitive behavioral intervention administered through a smartphone app
CBIA + TCC: Cognitive behavioral intervention administered through a Smartphone App + telephone conference call
CES-D: Center for Epidemiologic Studies Depression Scale
CSQ-8: Client Satisfaction Questionnaire
DMC: Data Monitoring Committee
DSM-5: Diagnostic and Statistical Manual of Mental Disorders
SCID-5-CV: Structured Clinical Interview for DSM-5 Disorders, Clinician Version
TCC: Telephone conference call
